# The level of using family planning methods and factors that influence the preference of methods in the Konya-Meram area

**DOI:** 10.4274/jtgga.2016.0180

**Published:** 2017-06-01

**Authors:** Zeynep Öztürk İnal, Hasan Ali İnal, Hasan Küçükkendirci, Ayla Sargın Oruç, Oğuzhan Günenç

**Affiliations:** 1 Departmant of Obstetrics and Gynecology, Konya Training and Research Hospital, Konya, Turkey; 2 Department of Public Health, Necmettin Erbakan University Meram Faculty of Medicine, Konya, Turkey; 3 Departmant of Obstetrics and Gynecology, Güven Hospital, Ankara, Turkey

**Keywords:** Family planning method, education level, financial status, reproductive period

## Abstract

**Objective:**

To determine the level of contraceptive method use and factors that influence the preference of method among women of reproductive age that live in Meram, the central district of Konya.

**Material and Methods:**

Parameters such as age, duration of marriage, number of pregnancies and births, socioeconomic status, education level, and preferred contraceptive method of women who presented to the family planning outpatient clinic of our hospital over a five-year period between January 1st, 2010, and December 31st, 2015, were recorded and evaluated.

**Results:**

The mean age of the women was identified as 31.57±8.14 years, the mean duration of marriage was 10.3±8.14 years, the mean number of births was 1.92±1.01, and the mean number of children was 1.83±0.90. Among the women in the study group, 65% were high school graduates, 88.92% had social security, and 82.84% were in the middle-income group according to their financial status. Only 31 patients were not married officially. It was observed that the most preferred method was intrauterine device (IUD), and the least preferred method was subcutaneous implant (SI). The use of IUD, oral contraceptives, and SI increased as the socioeconomic status and educational level improved (p<0.05).

**Conclusion:**

To ensure that women of reproductive age use effective family planning methods, the education levels and socioeconomic status of women must be improved.

## INTRODUCTION

Family planning is defined as the health care service that has the primary goal of improving maternal and child health, and preventing maternal and infant deaths, thereby enabling women of reproductive age to have as many children as they wish while providing adequate care (1). Family planning services are within the scope of “Basic Health Services” and its purpose is not to limit the number of individuals in the family but to provide couples with help and counseling about having children (2).

Maternal and infant mortality rates are parameters that are developmental indicators and these rates are higher in developing countries compared with developed countries. A gap of less than two years between births significantly affects maternal health and increases the maternal death rate by increasing the probability of high-risk pregnancy (3, 4). In societies with higher education levels and socioeconomic status, marriage, pregnancy and fertility occur at older ages, and consequently the need for contraceptive methods increases. Using contraceptive methods prevents young- and old-age pregnancies and high fertility, and as the spacing between births increases, the number of high-risk pregnancies decreases and the maternal death rate also drops (5). Couples are being provided with the information and services needed to protect women’s and children’s health, and families have been educated effectively about fertility for the last 40 years in our country.

The total fertility rate reflects the mean number of children a woman may give birth to during her reproductive period (15-49 years of age). According to the Turkey Demographic and Health Survey, the birth rate was 2.18 in 2014, and 2.14 in 2015 (6). In other words, the mean number of children a woman might give birth to during her reproductive period is 2.14. This shows that this is above the population renewal rate of 2.1. According to these data, approximately 75% of women of reproductive age use any one of the contraceptive methods, but unfortunately, the ratio of effective method use is only approximately 50% (6).

In this study, it was aimed to identify the level of contraceptive method use and factors that influenced the preference of method among women of reproductive age who lived in the central district of Meram in Konya.

## MATERIAL AND METHODS

This study was approved by the local ethics comitte of Konya Training and Research Hospital (reference number: 2016-10-01). The study was conducted in the Women’s Diseases and Birth Clinic’s Family Planning Polyclinic of Konya Training and Research Hospital over a five-year period between January 2010 and December 2015, and included 10,730 women who wanted to use a contraceptive method and gave their consent to participate in the study. After their gynecologic examination was performed, the women were asked to complete a survey that identified their descriptive characteristics such as age, duration of marriage, education level, socioeconomic status and social security, and fertility characteristics such as the number of pregnancies, births and miscarriages, and also included questions about the family planning method they used.

Statistical analyses were performed using SPSS 15.0 for Windows (SPSS, Chicago, IL, USA). The data studied were recorded as mean ± standard deviation, minimum-maximum. The Chi-square test was used for statistical analysis. Statistical significance was set at p<.05.

## RESULTS

The distribution of the age, education level, social security status, and socioeconomic status of the women included in the study are presented in Table 1. According to this, over the five-year period, 80% of the 10,730 women were aged 20 to 40 years. In the study group, 65% of the women were high-school graduates, 88.92% had social security, and 82.84% belonged to the middle-income group.

The mean age of the women was 31.57±8.14 years, and the mean duration of marriage was 10.03±6.71 years at the end of the study. The mean number of pregnancies was identified as 2.64±1.46, the number of births was 1.92±1.01, the number of miscarriages was 1.71±0.89, and the mean number of children was 1.83±0.90 (Table 2).

The distribution of family planning methods preferred by the women evaluated within the study is presented in Table 3. The most preferred method was intrauterine device (IUD) (37.38%), and this was followed by coitus interruptus (CI) (15.74%) and oral contraceptives (OCs) (15.65%), and the least preferred method was subcutaneous implant (SI) (3.17%).

In Table 4, the family planning methods used are presented in association with education levels. Women with primary school level education mostly preferred IUD and CI, whereas women with high-school or university level education mostly preferred IUDs and OCs. SI was mostly preferred by women who were university graduates (p<.05).

When the birth control method preferred by the patients was assessed in accordance with the socioeconomic status, it was observed that women with low and middle-income levels preferred IUD and CI, those with better income levels preferred OCs and IUD (p<.05). SI was mostly used as a family planning method by women with middle-level income (Table 5).

## DISCUSSION

In our study, it was aimed to identify the association between education level and socioeconomic status, and the contraceptive methods preferred by interviewing 10,730 women who presented to our hospital’s family planning polyclinic over five years between January 2010 and December 2015. The mean age of the women was 31.57±8.14 years, the duration of marriage was 10.03±6.71 years, the most preferred method was IUD, and the least preferred method was SI. It was identified that the use of effective modern methods, IUDs and OCs, increased as the education level and socioeconomic status of the women improved. It was identified that the use of IUDs and OCs increased as the education level and socioeconomic status of the women improved.

There are different data about the most preferred family planning methods in the existing literature. Kitapcıoğlu and Yanıkkerem (7) and Özdemir et al. (8) reported that IUDs (48.8%) were the most commonly used contraceptive method (48.8% and 50.7%), but Yıldırım et al. (9) reported condoms (39.1%) as the most commonly used contraceptive method.

In a study performed by Sak et al. (10) on the relationship between education level and contraceptive method preference, CIs (42.1%) were the most commonly used method and they identified that the use of IUDs and OCs increased as the level of education increased.

According to the data of the Turkey Demographic and Health Survey and the Institute of Population Studies, modern contraceptive methods are more preferred than conventional methods in Turkey today. The most common conventional method used was CI with a ratio of 25%, and the most common modern method used was IUD (11, 12).

The study conducted by Kaya et al. (13) with 303 women of reproductive age to demonstrate the level of contraceptive method use showed that the most common methods were CIs (23.1%), IUDs (21.5%), condoms (19.8%), and OCs (13.9%), and it was shown that the ratio of modern and effective contraceptive method use increased as the socioeconomic status improved.

In our study, the most preferred contraceptive methods were IUDs (37.38%), CIs (15.74%), OCs (15.65%), condoms (12.8%), depot progesterone (9.03%), bilateral tubal ligation (6.25%), and SIs (3.17%), respectively.

The conventional CI method was the second most common method used in our study. Although its ease of use and lack of cost appear to be the advantages of this method, which was once widely used by most societies, its chance of success is rather low in comparison with other methods because it effectiveness depends on the motivation and adjustment of couples.

IUDs are one of the preferred modern methods and its ease of use, long-lasting effect, high-reliability rates, the possibility of use during the breastfeeding period, and the fast return of fertility once the method is abandoned are among its very important advantages; conditions such as irregular menstruation are its disadvantages.

OCs are another modern and effective method with similar advantages to IUDs and have the additional advantage of not causing irregular menstruation. Its only disadvantage may be the probability of forgetting to take the pill. This negative aspect may be counteracted by motivation to some extent (14). The use of OCs was in third place with a ratio of 15.65%.

In parallel with the increase of the frequency of sexually-transmitted diseases, the use of condoms has also increased. In a study conducted by Inal (15), the rate of condom use in our country was reported as 5%, and it was reported as 11.3% in the study conducted by Çınar et al. (16). In our study, the ratio of condom use was reported as the fourth most common method used, similar to the second study.

In the study conducted by Civi and Bodur (17) in 1991 in Konya, which was performed by interviewing 265 women, it was identified that 78.9% of women were protected from pregnancy and that 79.4% of these women used an effective method. The use of modern and effective contraception methods increased up to 85% in our study.

We observed that IUDs were the most preferred method of contraception in this particular region. This might be explained to some extent with their easy access and being covered by the social security system. In Turkey, Family Health Centers provide free IUDs; their installation and all gynecologic examinations are free in these centers. In contrast, OCs are not paid for by the social security system.

The importance of informing couples must not be forgotten to ensure that contraceptive methods do not fail. Even though audiovisual media has an important role in providing information and raising awareness, health care providers must also be devoted to education and counseling. Such an education and counseling service is most accessible and appropriate during the follow-up periods before and after giving birth (18). Women are particularly inclined towards contraceptive methods during the postpartum period and more sensitive and effective efforts made by health care providers during this period would provide benefit.

Our study has the largest number of patients among the related studies in the current literature. The geographic region in which the participants resided represents typical Anatolia. Therefore, the results of this study may reflect Turkey in general.

In conclusion, choosing effective and reliable contraceptive methods is directly proportional to the education level and socioeconomic status of women. To ensure that women of reproductive age have higher ratios of access to contraceptive methods in addition to raising awareness via audiovisual media, health care workers should also be more sensitive and dedicated. Achieving healthy pregnancies by means of modern and effective contraceptive methods will help drop the maternal and infant death rates, which are among the key developmental indicators. In addition, studies directed to improving education levels and socioeconomic status of women of reproductive age are also required.

## Figures and Tables

**Table 1:**
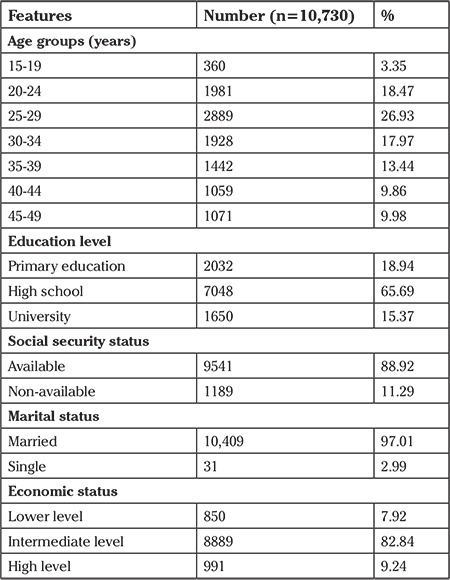
The distribution of women according to the demographic features

**Table 2:**
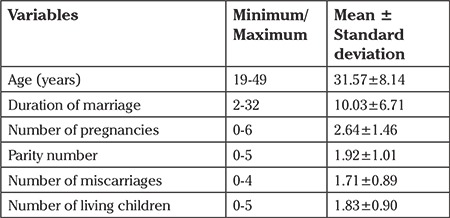
The fertility data of the women

**Table 3:**
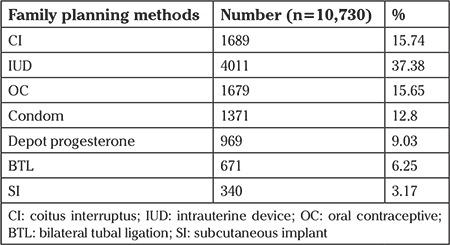
The distribution of family planning methods used by the women

**Table 4:**
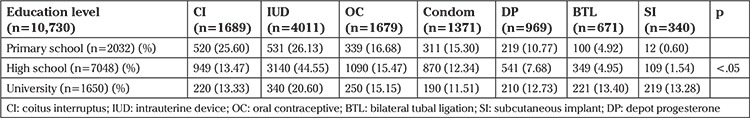
The distribution of the birth control methods preferred by the women with respect to education level

**Table 5:**
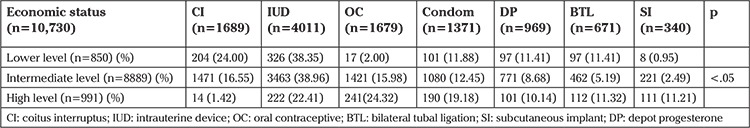
The distribution of birth control methods preferred by the women with respect to their socioeconomic status
